# P-71. Assessment of Oral Third-generation Cephalosporins Step-Down Therapy for UTI-Related Bacteremia

**DOI:** 10.1093/ofid/ofaf695.300

**Published:** 2026-01-11

**Authors:** London Umejiaku, Stefanie Stafford, Todd Price, John Badalamenti, Thomas Roduta

**Affiliations:** Detroit Receiving Hospital , Detroit, MI; Memorial Hermann Health System, Houston, Texas; Memorial Hermann Health System, Houston, Texas; John Hopkins Hospital, Baltimore, Maryland; Memorial Hermann Health System, Houston, Texas

## Abstract

**Background:**

Recommended step-down oral therapies for urinary-origin gram-negative bacteremia are fluoroquinolones (FQ) and sulfamethoxazole-trimethoprim (SMX/TMP). Safety concerns associated with these options include their side effects and pathogen resistance. Oral third-generation cephalosporins (TGC) have increasing data supporting use as a step-down therapy. Here we compared the efficacy of oral TGCs to the standard of care (FQ or SMX/TMP) for the treatment of urinary-origin, uncomplicated gram-negative bacteremia.

**Methods:**

An observational retrospective chart review was performed on adult patients who presented to a large hospital system from January 2019 to December 2023. Included patients received an oral TGC, FQ or SMX/TMP, and had urine and blood cultures positive for *Escherichia coli*, *Klebsiella* spp., or *Proteus* spp. Excluded patients had polymicrobial urine or blood cultures, non-urinary source bacteremia, received multiple non-empiric antibiotics concomitantly, less than 72-hour oral therapy, or had urethral catheters.

**Results:**

For the cohort overall (n= 161), *Escherichia coli* accounted for 82%, followed by *Klebsiella sp.* (12.4%), and *Proteus sp*. (5.6%). The primary outcome of 30-day bacteremia recurrence, showed no difference between the TGCs group (n=47) at 0 events and standard-of-care group (n=114) at 0 events, (p=1). Secondary and safety endpoints showed a median (IQR) for length of stay in days of 4.7 (3.0-6.4) vs 3.8 (2.9-6.3), (p=0.31); length of inpatient therapy in days of 4 (3-6) vs 4 (3-5), (p=0.56); 30-day UTI recurrence was 1 vs 5 events, (p=0.67); 30-day all-cause mortality were 0 vs 2 events, (p=1). Patients in the TGCs group had a median age of 73 (62-81) vs 61 (44.75-80), (p=0.0049); median comorbidity score of 4 (2-5) vs 2 (0-2), (p=0.006); and median length of total treatment duration in days of 8 (4-14) vs 14 (10.5-17), (p=0.00001). The primary TGC utilized was cefdinir at 83% with cefpodoxime at 17%.

**Conclusion:**

TGCs produced low rates of bacteremia recurrence, comparable to those found in patients treated with the standard-of-care. These results support an increased investigation of TGCs use for oral step-down therapy in the setting of gram-negative bacteremia with a urinary source for the identified organisms.

**Disclosures:**

All Authors: No reported disclosures

